# Criteria for the assessment of analyser practicability

**DOI:** 10.1155/S1463924693000227

**Published:** 1993

**Authors:** C. Biosca, R. Galimany

**Affiliations:** Sociedad Española de Química Clínica LLançà 53, Baixos 3a Barcelona 08015 Spain

## Abstract

This article lists the theoretical criteria that need to be considered to assess the practicability of an automatic analyser. Two essential sets of criteria should be taken into account when selecting an automatic analyser: ‘reliability’ and ‘practicability’. Practibility covers the features that provide information about the suitability of an analyser for specific working conditions.

These practibility criteria are classsified in this article and include the environment; work organization; versatility and flexibility; safely controls; staff training; maintenance and operational costs.


Journal of Automatic Chemistry, Vol. 15, No. 5 (September–October 1993), pp. 167–170

### Criteria for the assessment of analyser practicability

**Prepared by C. Biosca and R. Galimany for the Spanish Society of Clinical Chemistry: Scientific Committee. Instrumentation Commission***

*Sociedad Española de Química Clínica, LLança, 53, Baixos 3a, 08015 Barcelona, Spain*

*This article lists the theoretical criteria that need to be considered to assess the practicability of an automatic analyser. Two essential sets of criteria should be taken into account when selecting an automatic analyser: 'reliability' and 'practicability'.* Practibility *covers the features that provide information about the suitability of an analyser for specific working conditions.*

*These practibility criteria are classsified in this article and include the environment; work organization; versatility and flexibility; safety controls; staff training; maintenance and operational costs.*

### Introduction

This paper describes the theoretical criteria that need to be considered in order to assess the practicability of an automatic analyser for eventual use under specific working conditions. The criteria to be considered in selecting an analyser are reliability and practicability.

*Reliability* can be defined as the ability an analyser has to maintain a good analytical quality (imprecision, inaccuracy, shift etc.) on a long-term basis.

*Practicability* includes a number of qualities that provide information about the whole range of possibilities the analyser has under the specific working conditions of a given laboratory.

An analyser will be selected on the basis of manufacturers' technical information, the experiences of other users and published evaluations. A good evaluation will cover all aspects of analysis, both favourable and unfavourable, and provide potential users with the information that will allow them to select the instrument that best suits their specific working requirements.

'Practicability' criteria can be classified as follows:

1. Environment.
2. Work organization.
   - 2.1. Start operations.
   - 2.2. Sample handling.
     - 2.2.1. Display of samples and type of samples.
     - 2.2.2. Sample identification.
     - 2.2.3. Aspiration of samples.
   - 2.3. Reagents.
   - 2.4. Reaction trays.
   - 2.5. Processing speed.
   - 2.6. Methods.
   - 2.7. Information processing.
3. Versatility and flexibility.
4. Safety controls.
5. Staff training.
6. Maintenance.
7. Operational costs.

### 1. Environment

Analyser: size and weight.

Additional parts (printer, bar-code reader, screen and keyboard, computer etc.); size and weight.

Specific features for electric power outlets.

Need for power stabilizer.

Need for water (distilled, de-ionized) outlet; and consumption per hour.

Air-conditioning and environmental temperature for proper functioning.

Need for drains: reagent removal, automatic or manual.

Compressed air.

Noise, in decibels, from the analyser and printer (within permitted levels).

### 2. Work organization

### 2.1. *Start operations*

Time required by the analyser to start any kind of analytical process.

Eventual automatic programming of start operations.

Length of time devoted daily to analyser tuning operations (before the analysis itself).

### 2.2. *Sample processing*

Working with one sample at a time, or by batches or both. Availability: for example 24 hours' operation.

#### 2.2.1. *Sample display and type of samples*

Level of difficulty and accessibility of sample load.

Protection against sample evaporation.

Processing of microsamples.

Possibility of carrying out analysis on an emergency basis: special sample holder; conventional sample holder (sample processed with the usual routine); time needed

* Members of the Commission are: M. J. Alsina, A. Alumà, C. Armenter, C. Biosca, J. Bravo, M. Doladé, J. Ferré, R. Galimany and M. Martinez.

0142-0453/93 $10.00 © 1993 Taylor & Francis Ltd.

for processing samples on an emergency basis and interference with the previously planned workload.

Processing of samples of urine and other biological fluids.

Use of primary tube, tubes, cups and microcups on the same sample holder.

2.2.2. *Sample identification*

Without bar-code.
Label with bar-code on sample holder.

Label with bar-code on primary tube. Use of labels with bar code: with the operator taking part in the reading (with optic laser, scanner, etc.); without the operator taking part in the reading: reader built into the sample holder that identifies the sample, versus the optic laser which is located in the analyser (this is the most reliable and safest kind of identification).

Assessment of sample identification system.

Assessment of deficiencies in bar-code reading.

Is it essential to have the label on the tube; is the proper position key to the proper reading; to what extent does label decay matter etc.?

2.2.3. *Sample aspiration*

Sample aspiration from the cup.

Sample aspiration straight from the primary tube; eventual use of different sizes.

Eventual sample aspiration from a closed primary tube.

Prerequisites for the use of primary tubes.

Effectiveness of fibrine detectors and sample level detectors.

Sample dead volume.

Washing of sample pipette (inside and outside) to prevent sample/sample, reagent/reagent or both kinds of contamination.

2.3. *Reagents*

Needs preparation or is ready-to use.

Storage in the reagent compartment of the analyser, cooled or not, or outside the reagent compartment.

Reagent compartment: capacity; versatility in display of reagent volumes; eventual preparation of different volumes according to requirements.

Mandatory use of reagents with very short expiration spans.

Easy introduction of reagent while the analyser is at work.

Eventual stock chamber within refrigerated reagent compartment.

Precise knowledge of available reagents, expiration and calibration dates.

Management of reagent stocks by the analyser, reagent volume indicator.

Eventual use of a single reagent or a combination of several reagents.

2.4. *Reaction trays*

Disposable or retrievable after washing in the analyser.

Cleansing control of cuvettes before their use. Automatic elimination of defective cuvettes.

Proposed rate of change of cuvettes.

2.5. *Processing speed*

Processing speed, number of measurements/hour under routine conditions.

Influence of number and type of constituents on processing speed.

Time required to obtain a result from the introduction of samples.

Time required to obtain a result on an emergency basis.

Causes of delay: cleaning circle or tray exchange, priming of solvent, priming with reagents, etc.

Time devoted to stopping the sampling process while working on a routine basis in order to calibrate or refill with reagents.

Stopping: immediate availability without a time delay.

2.6. *Methods*

Combination of available measuring systems in analyser (spectrometry, nephelometry, turbidimetry, potentiometry or other).

2.7. *Information processing*

Assess data entered through keyboard and their difficulty.

Editing: one patient at a time, by a series of results, or both.

Possibility of programming the edition format of results.

Possibility of visualizing results on the screen even when incomplete.

Ability to correct and validate patient's results on the screen.

Possibility of adding the results of other measurements to the final report.

Eventual search of a patient on the analyzer file by laboratory number, identification number or other demographic data, in order to review him on the screen or re-edit it.

Possibility of fast editing of a patient's report if required tests are performed before those of a previous patient.

Filing capacity (patients and results).

Possibility of setting up a bidirectional connection with the central computer. Transmission speed.

### 3. Versatility and flexibility

Open analytical system: free choice of reagents; ability to adapt reagents and methods not initially anticipated.

Closed analytical system: possibility of a dialogue between the manufacturer and the user about methods, reviews and program changes;

Use of methods recommended by international clinical chemistry organizations.

Number of reagents per test.

Ability to choose wavelengths.

Monochromatism, bichromatism or polychromatism.

Sample, reagent and diluent volumes: choice possibilities.

Maximum and minimum reaction volumes.

Programmable incubation time.

Free choice of measuring times.

Number of points for programmable reading.

Measuring modes (continuous, end point, linear, non-linear etc.).

Number of reagents simultaneously available.

Use of one or more cuvettes per determination.

Change in speed with the use of one or two reagents.

### 4. Safety controls

Easy access to analytical modules.

Automatic checking of sample volume required to perform the analysis.

Checking the existence of sample tube.

Error signals if no sample or tube are detected.

Whenever a tube is not processed, determinations remain on a waiting list without introducing delays.

Protection against contamination.

Reaction temperature control.

Lamp status control.

*Calibration control*

Display of calibrators and their preservation.

Calibration frequency.

Calibration control by the operator.

Calibration on every change of reagent lot.

Number of possible dosages for each calibrator and estimates in order to obtain the final value.

Number of calibrators, single or simultaneous use.

Automatic calibration at a pre-set frequency.

Edition in absorbance units or other units of calibration values.

Selective calibration.

Time spent in calibration.

*Quality control*

Quality control programme, algorithms available.

Control alarms: by the result, instead of result.

Blocking alarm, sample holder stops the ongoing process only or all of them.

Nonblocking alarm.

Acceptance decided by the operator.

Absorbance control of reaction blanks.

Printing of results with alarm signal if they go beyond linearity limits.

Temperature control: alarms and messages.

Continuous control of measurements.

Enzyme hyperactivity: automatic correction.

Ability to correct interferences due to turbidity, haemolysis and bilirubin.

Messages in pathologic results from patients.

Messages due to errors in sample processing.

Reference values. Differences based on age and sex.

Possibility of performing automatic dilution of a sample, or repeating a previous determination, or both.

Possibility of repeating samples with the same identification (repeating only what matters) and storing the accepted results on the patient's file.

Re-editing results.

Type of statistical display of reports about analysis performed, reagents consume, etc.

In case of the analyser stopping suddenly, how does this affect the routine work? Time necessary to restart.

Ability to enter requests, validation and editing of results if there is a computer breakdown.

Percentage of repetitions per day and per constituent.

### 5. Staff training

Time required to learn to work with the system.

Time required to learn to solve problems.

User friendliness.

Information systems for the user: amount of information given to the user on screen; system status indicators on screen, for example: stop, on, alarms and error codes.

Access to different programmes and menus during sample processing.

Explanation of error codes available on the screen.

Display and contents of specifications manual; easy access; methodological information concerning endogenous and exogenous interferences for the different analytical procedures.

### 6. Maintenance

Time required for installation.

Pre-programmed cleaning; length of time needed for cleaning.

Time required for daily, weekly and monthly maintenance.

Time needed for other types of maintenance.

Regular revision of programmes.

Possibility of programme expansion, as well as changes and subsequent improvements.

After sale technical service.

Other working controls; self-diagnosis programme.

Frequent breakdown (ask the users).

Speed with which the technical service responds when the analyser breaks down or stops.

### 7. Operational costs

Number of operators required.

Materials required to operate, for example: disposable trays; reagents; and detergents.

Spare parts needed, for example pipettes; lamps; valves; connectors; tubes; mechanical parts; miscellaneous.

Tools and other supplies needed.

### Bibliography

*Instrumentation en biochimie clinique* (Commission Instrumentation: Monographie de la Société française de Biologie clinique).

Expert Panel on Instrumentation of the International Federation of Clinical Chemistry, Decision criteria for the selection of analytical instruments used in clinical chemistry, *Journal of Automatic Chemistry*, **2**, 20–32 (1982).

